# Ultrasensitive Human Urinary Albumin Detection via Composite Nanohydrogels

**DOI:** 10.3390/mi17040409

**Published:** 2026-03-27

**Authors:** Özge Altıntaş, Fatma Yılmaz, Elif Serra Taş, Adil Denizli

**Affiliations:** 1 Department of Chemistry, Hacettepe University, 06800 Ankara, Turkey; 2 Department of Chemistry and Chemical Processing Technologies, Bolu Abant İzzet Baysal University, 14900 Bolu, Turkey; 3 Department of Molecular Biology and Genetics, Başkent University, 06790 Ankara, Turkey

**Keywords:** albumin, C-Dots, molecular imprinting technology, nanohydrogel, biomolecule detection

## Abstract

Albumin is an important biomarker in biological fluids and plays a critical role, particularly in the diagnosis of renal dysfunction. Therefore, the sensitive detection of low concentrations of albumin in urine is of great importance. In this study, a composite nanohydrogel modified with carbon dots has been developed for the selective detection of albumin from human urine. The composite nanohydrogels were synthesised using a molecular imprinting technique specifically designed to recognise albumin. Characterisation studies were conducted using ZetaSizer, SEM, EDX, CLSM and ATR-FTIR methods. The albumin-binding capacities of the carbon dots (C-Dots) and synthesised composite nanohydrogels were evaluated using fluorescence spectroscopy. The effects of different concentration conditions on binding efficiency were systematically investigated. Selectivity studies have shown that albumin-imprinted nanohydrogels can detect target molecule albumin four times more selectively than competitive molecules, Hb and IgG. Imprinting efficiency was estimated by comparing the signals of albumin obtained from non-imprinted and albumin-imprinted composite nanohydrogels. Finally, artificial urine samples mimicking real biological environment conditions were examined to evaluate matrix effect on the albumin detection. The repeatability and long-term stability of albumin detection, performed with four consecutive and six-month measurements, was evaluated using the %RSD value, confirming that the albumin determination performance was maintained.

## 1. Introduction

Protein levels in biological fluids are used as important biomarkers in the diagnosis and monitoring of many diseases [1]. Among these, albumin is the most abundant protein in human serum, with a molecular weight of approximately 66.5 kDa, and plays a role in regulating osmotic pressure and transporting many drugs, ions and hormones [2]. Clinically, the determination of microalbuminuria levels plays a critical role in the early detection of kidney dysfunction, such as diabetic nephropathy, hypertensive nephropathy and glomerular diseases. Kidney damage in patients with diabetic nephropathy leads to leakage of small amounts of albumin into the urine, typically in the range of >30–300 μg/mL, a condition clinically characterised as microalbuminuria [3]. Therefore, highly sensitive and selective separation techniques are required. Recent advances in nanotechnology and advanced material design have enabled the development of separation systems for the identification of biological analytes [4]. In this context, nanohydrogels offer advantages such as high surface area, controlled permeability, biocompatibility, swelling behaviour and sensitivity to target molecules by combining both hydrogel and nanomaterial properties. Nanohydrogels are special hydrogels consisting of three-dimensional polymeric networks with water-holding capacity in the nanometre range (1–100 nm). These materials combine both hydrogel properties (swelling, biocompatibility, flexibility) and nanoscale advantages (high surface area, controlled release and cell penetration) [5]. Furthermore, due to their ability to respond to environmental stimuli (ionic strength, temperature and pH) through volumetric changes, these materials are gaining increasing interest in bioseparation [6], drug delivery [7], tissue engineering [8] and biosensor technologies [9]. However, for nanohydrogels to exhibit high specificity toward target proteins, they must possess not only physical interactions but also structural recognition mechanisms [10]. Molecular imprinting technology offers an extremely powerful approach to meet this requirement [11]. Molecular imprinting technology is based on the principle of incorporating a specific target molecule (template) into the structure during the polymerisation process and subsequently removing it from the environment [12]. As a result of this process, a polymer matrix is obtained that contains unique recognition sites complementary to the size, geometry and functional groups of the template molecule. These structures contain artificial recognition sites capable of forming highly selective bonds with the template molecule, making them an ideal platform for the selective separation of low-concentration analytes, particularly in complex biological environments [13]. Molecularly imprinted polymers (MIPs) are widely used in sensor technologies [14], drug delivery [15], environmental analysis [16] and bioselective detection [17] applications as durable and reusable systems exhibiting antibody-like behaviour. When integrated with nanohydrogels, these systems offer faster diffusion, enhanced surface interaction and higher binding kinetics due to their high-water retention capacity and three-dimensional porous structure [18,19]. In this context, molecularly imprinted nanohydrogels provide both target molecule-specific selectivity and significantly enhance functionality and efficiency in biological applications [20]. Carbon-based nanostructures have attracted significant attention in recent years in the fields of nanotechnology and biomaterials, with carbon dots (C-Dots) particularly coming to the fore in biomedical applications [21,22]. These fluorescent structures, typically smaller than 10 nanometres in size, are highly appealing due to their high biocompatibility, low toxicity and excellent optical properties [23]. Compared to metal-based quantum dots, their greater safety makes them a preferred alternative in biological systems [24]. The strong fluorescent properties of C-Dots make them important tools in bioimaging, while their high resolution and stability in aqueous environments offer a major advantage for biological applications [25]. In this context, C-Dots-modified nanohydrogels offer ideal hybrid structures, particularly for bioselective separation systems [26]. The integration of C-Dots into the nanohydrogel structure not only provides structural support but also enhances the overall performance of the system by creating active regions that facilitate interaction with target molecules [27,28].

In this study, we developed composite nanohydrogel structures based on molecular imprinting technology for the selective binding of albumin molecules. These developed structures provide high selectivity and binding capacity by creating specific recognition sites for albumin within the hydrogel matrix. Additionally, C-Dots integrated into the nanohydrogel structure have improved the surface properties of the nanohydrogels and provided analytical tracking capabilities through their fluorescent properties. The nano-scale porous structure and large surface area have increased the system’s adsorbent performance, supporting its efficiency in biomolecule detection processes. C-Dots-modified non-imprinted and albumin-imprinted nanohydrogels were characterised by zeta size measurements (ZetaSizer), attenuated total reflectance-Fourier transform infrared (ATR-FTIR), confocal laser scanning microscopy (CLSM), energy dispersive X-ray spectroscopy (EDX) and scanning electron microscopy (SEM) analyses. Additionally, the effects of albumin concentration on the sensitive detection capability of the synthesised C-Dots, non-imprinted and albumin-imprinted nanohydrogels were investigated using fluorescence spectroscopy analysis. Additionally, selectivity studies were conducted with competitor molecules similar to albumin and the selectivity of composite nanohydrogels towards albumin was analysed. In addition, fluorimetric analyses were performed in artificial urine samples that mimic the ionic and molecular composition of human urine in order to verify the applicability of the developed system in real samples. This study presents a selective platform for protein detection in biomedical and pharmaceutical applications.

## 2. Experimental

### 2.1. Chemicals

Leucine methacrylamide (MALM) functional monomer was supplied by Nanoreg company (Ankara, Turkey), while methylenebisacrylamide (MBAA), hydroxy ethyl methacrylate (HEMA), ammonium persulfate (APS), bovine haemoglobin (Hb) and human serum immunoglobulin G (IgG) were obtained from Sigma-Aldrich Corporation (MO, USA). Human serum albumin (HSA) was purchased from Bayer Corporation (Elkhart, IN, USA). PBS tablets, NaCI, NH_4_Cl, CaCl_2_.2H_2_O, Na_2_HPO_4_, urea, creatinine and other chemicals of analytical purity were obtained from Merck (Darmstadt, Germany).

### 2.2. Synthesis of C-Dots

C-Dots were synthesised by a bottom-up microwave-assisted hydrothermal method [29]. 0.252 g ethylenediamine and 0.4203 g citric acid were completely dissolved in 80 mL of ultrapure water to obtain a homogeneous solution. This solution was transferred to a Teflon-coated stainless-steel autoclave and subjected to hydrothermal treatment at 160 °C for 4 h. After the reaction was completed, the system was cooled to room temperature, and then the reaction mixture was centrifuged at 14,100× *g* for 20 min (Eppendorf MiniSpin^®^ Series Centrifuge, Hamburg, Germany) to remove any large aggregates that might form. The supernatant was passed through a membrane filter with a pore diameter of 0.22 µm to remove residual particles. The resulting solution was purified by dialyzing against ultrapure water for 24 h using a dialysis membrane with a molecular weight cutoff value of 500 Da to remove unreacted small molecules and impurities.

### 2.3. Synthesis of Composite Nanohydrogels

**Figure 1:**
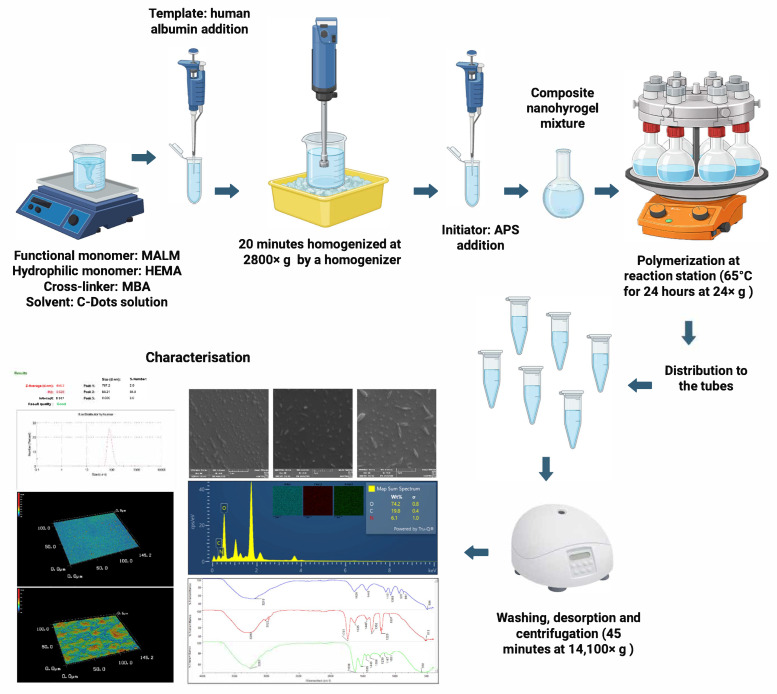
Schematic diagram of preparation of composite nanohydrogels.

Nanohydrogels modified including C-Dots were synthesised according to the recipe with minor modifications [30]. An amount of 50 mL of polymerization solution containing 50 mM total monomer concentration was prepared in ultrapure water to include the quantitative C-Dots solution. MBAA was utilised as a crosslinker at 2% concentration, while MALM served as a functional monomer at 25% and HEMA acted as a hydrophilic monomer at a 75% ratio (*w/w*). The molar ratio of template molecule albumin to the functional monomer MALM was set at 1:250 (µmol/mol). The synthesis steps of composite nanohydrogels are schematically shown in Figure 1.

At first, monomer phase was homogenised at 2800× *g* by a homogenizer (T10, Ika Labortechnik, Staufen im Breisgau, Germany) for 20 min to obtain an emulsion. After these components were poured into the three-mouthed, round-bottomed flask containing C-Dots solution, the mixture was heated to 65 °C and then 20 mg of ammonium persulfate (APS) was added as the initiator. The entire process was carried out at 65 °C with mechanical stirring at 24× *g* for 24 h on the polymerization system (Radleys, Carousel 6 Plus Reaction Station, Saffron Walden, UK). The non-imprinted nanohydrogels were also synthesised with the same procedure except adding the albumin. The non-imprinted and albumin-imprinted nanohydrogels washed with ethanol, ethanol–water mixture at a ratio of 50:50 (*v/v*) and water in respectively using centrifugation at 14,100× *g* for 45 min (dorf MiniSpin^®^ Series Centrifuge, Hamburg, Germany). As a desorption agent, 0.1 M NaCl solution was used for the removal of albumin from the albumin-imprinted nanohydrogels. The desorption process was continued until no absorbance observation was reported at 280 nm by UV-Visible Spectrophotometry (Thermo Fisher Scientific, Genesys 10s UV-Vis, Waltham, MA, USA). Fluorimetric measurements were also performed to verify the removal of the template. Fluorimetric measurements of non-imprinted nanohydrogels and template-removed albumin-imprinted nanohydrogels showed similar results. These results that the template was successfully removed from the albumin-imprinted nanohydrogels. Figure S1 shows the fluorimetric measurements of non-imprinted nanohyrogels and template-removed albumin-imprinted nanohydrogels. After washing steps, nanohydrogels were dispersed in ultrapure water and kept at 4 °C until use.

### 2.4. Characterisation Analysis

Characterisation analyses were performed to evaluate the size distribution, chemical structure and surface morphology of C-Dots and synthesised composite nanohydrogels. ZetaSizer Nano-ZS (Malvern Instrument Company, Malvern, UK) device was used to determine particle sizes and size distribution of particles. The morphological properties and surface topography of the nanostructures were examined in detail using scanning electron microscopy (SEM, Tescan, GAIA3 + Oxford Xmax 150 EDS, Brno, Czechia). At the same time, the elemental compositions of the nanohydrogels were determined using energy dispersive X-ray (EDX, Tescan, GAIA3 + Oxford Xmax 150 EDS, Czechia) during this analysis. confocal laser scanning microscopy (CLSM, Keyence VKX 100 Laser Confocal Non-Contact Profilometer, Itasca, IL, USA) was used for a more detailed evaluation of the three-dimensional surface properties and homogeneity of albumin-imprinted nanohydrogels. Furthermore, the chemical characterisation of the functional groups on the synthesised structures was performed using attenuated total reflectance-Fourier transform infrared spectroscopy (ATR-FTIR, Thermo Fisher Scientific, Nicolet iS10, Waltham, MA, USA) and the effects of the molecular imprinting process at the chemical level were confirmed.

### 2.5. Optical Analysis

In this study, the optical behaviour properties of C-Dots and non-imprinted and albumin-imprinted nanohydrogels were examined in detail under different albumin concentrations (0, 30, 100, 200 and 300 ppm). In this context, fluorescence spectroscopy (Shimadzu RF-5301 PC, Kyoto, Japan) was used to evaluate the fluorescent properties. Additionally, measurements were taken using UV-visible spectrophotometry (Thermo Fisher Scientific, Genesys 10s UV-Vis, USA) to create a calibration curve at 280 nm for different albumin concentrations and to measure the absorbance values of C-Dots. The measurements aimed to reveal both the natural fluorescent responses of C-Dots and the optical interactions of C-Dots-modified nanohydrogels with albumin. Since the albumin and C-Dots concentrations used in the study given high absorbance values, the samples were diluted at a ratio of 1:200 (*v/v*) to ensure that the measurements remained within the linear range of the instrument and then measurements were taken. The fluorescence data obtained at different albumin concentrations determine the degree of interaction of these systems with the target molecule and provide important clues about the binding capacity properties of the nanohydrogels. In obtaining the fluorescence spectra, C-Dots solutions were diluted at a ratio of 1:1000 (*v/v*), then 500 µL (9.0 mg·mL^−1^) of this diluted solution was taken and 1000 µL of albumin solutions prepared in PBS buffer in the range of 0–300 ppm was added. After the mixtures showed a homogeneous distribution, fluorescence measurements were taken. For albumin-imprinted and non-imprinted composite nanohydrogels, the nanohydrogels were first centrifuged at 14,100× *g* for 45 min to precipitate. 500 µL of non-imprinted nanohydrogel (980 mg·mL^−1^) and 500 µL of albumin-imprinted nanohydrogel (990 mg·mL^−1^) remaining in the precipitated phase were separately added to 1000 µL of albumin solution prepared in PBS buffer within the range of 0–300 ppm. After the mixtures showed a homogeneous distribution, fluorescence measurements were performed (λ_exi_: 350 nm; Slit Width: EX: 5.0 nm, EM: 5.0 nm).

### 2.6. Selectivity Studies

To evaluate the selectivity behaviour of the developed composite nanohydrogels towards the template molecule albumin, selectivity analyses were performed using fluorescence spectroscopy (Shimadzu RF-5301 PC, Japan). In this context, the interactions of albumin-imprinted and non-imprinted composite nanohydrogels with other molecules having similar molecular weight and isoelectric point to albumin were investigated. Hb and IgG were used as control molecules. In the analyses, solutions prepared with HSA, Hb and IgG at 300 ppm concentration were interacted with composite nanohydrogels and the resulting changes in fluorescence intensity were compared. These measurements were performed to reveal the binding behaviour of each nanohydrogel type towards different molecules. For albumin-imprinted and non-imprinted composite nanohydrogels, the nanohydrogels were first centrifuged at 14,100× *g* for 45 min to precipitate. 500 µL of non-imprinted nanohydrogel (980 mg·mL^−1^) and 500 µL of albumin-imprinted nanohydrogel (990 mg·mL^−1^) remaining in the precipitated phase were separately added to 1000 µL of HSA, Hb and IgG solutions prepared in PBS buffer within the range of 0–300 ppm. After the mixtures showed a homogeneous distribution, fluorescence measurements were performed.

The selectivity coefficients (k) for the albumin molecule and competitor molecules (Hb and IgG) are calculated using the following equation:

(1) $$k=\frac{I_{template}}{I_{competitor}}$$

where I_template_ represents the highest intensity value for the HSA molecule and I_competitor_ represents the highest intensity values for the competitor molecules, Hb and IgG. The relative selectivity coefficient (k′) is used to consider the effect of imprinting on albumin selectivity can be determined from the following equation:

(2) $$k^{\prime }=\frac{k_{imprinted}}{k_{non-imprinted}}$$

where k_imprinted_ is the relative selectivity coefficient of the imprinted composite nanohdyrogel carrier, k_non-imprinted_ is the relative selectivity coefficient of the non-imprinted composite nanohdyrogel carrier.

Imprinting efficiency (IE) is an important parameter that quantitatively expresses the degree of success of the molecular imprinting process for the target molecule. This concept is based on comparing the signals given by imprinted and non-imprinted structures to the target molecule. In this study, since the albumin molecule was used as a template during the molecular imprinting process, albumin-specific recognition cavities are formed within the polymer matrix. These specific cavities enable selective binding by exhibiting shape, size and functional group compatibility with albumin. I_imprinted_ is the highest intensity value of the imprinted composite nanohydrogel carrier, I_non-imprinted_ is the highest intensity value of the non-imprinted composite nanohydrogel carrier. IE albumin signals obtained from imprinted and non-imprinted nanohydrogels were calculated using the following equation:

(3) $$IE=\frac{I_{imprinted}}{I_{non-imprinted}}$$

### 2.7. Repeatability and Long-Term Stability Studies

Repeatability and long-term stability studies were conducted to evaluate whether the developed analytical system provides consistent and reliable results in successive measurements performed at short and long intervals under the same experimental conditions. This analysis is one of the most important criteria revealing the analytical performance of nanohydrogel-based sensing systems and reflects the stability, measurement accuracy and reusability potential of the system. In this study, the repeatability of albumin-imprinted composite nanohydrogels was investigated by performing four consecutive fluorescence measurements with a 300 ppm albumin solution and six-month long-term stability analyses using the same nanohydrogel sample. The relative standard deviation (%RSD) value was calculated using the fluorescence intensities obtained after each measurement. The %RSD value expresses the deviation between measurements as a ratio to the average signal and is used as a quantitative indicator of the measurement accuracy of the system.

(4) $$\%RSD=\frac{standard\;deviation}{average\;value}\times 100\%$$

### 2.8. Analysis in Artificial Urine Samples

To evaluate the performance of the developed hybrid nanohydrogels in systems mimicking real biological environment conditions, experimental studies were repeated using artificial urine samples. The artificial urine environment was prepared considering the ionic components, pH conditions and organic contents naturally found in human urine; thus, the selectivity and binding behaviour of the developed system within the biological matrix was observed. The analyses were performed using fluorescence spectroscopy (Shimadzu RF-5301 PC, Japan). This allowed the fluorescent responses of the hybrid nanohydrogels to the presence of albumin in the artificial urine environment to be monitored and the sensitivity and selectivity of the system to be evaluated. The primary aim of these analyses was to demonstrate that the composite nanohydrogels used for albumin determination can function effectively and exhibit remarkable stability not only in buffer solutions but also in biologically relevant complex environments. Thus, the clinical applicability and analytical reliability of the synthesised systems were evaluated. The artificial urine sample was prepared by simulating the natural urine according to the report prepared by the Reference Materials and Measurements of the European Commission [31]. With this formula, called AU-Siriraj, artificial urine is obtained by mixing 0.034 g uric acid, 2.427 g urea, 0.090 g creatinine, 0.450 g KCl, 0.634 g NaCl, 0.161 g NH_4_Cl, 0.297 g Na_3_C_6_H_5_O_7_·2H_2_O, 0.034 g NaHCO_3_, 0.003 g NaC_2_O_4_, 0.089 g CaCl_2_·2H_2_O, 0.100 g MgSO_4_·7H_2_O, 0.100 g NaH_2_PO_4_·H_2_O, 0.011 g Na_2_HPO_4_ and 0.258 g Na_2_SO_4_ in 200 mL. It is prepared by dissolving it in deionized water. The pH of the solution is adjusted to be within the physiological range. For albumin-imprinted and non-imprinted composite nanohydrogels, the nanohydrogels were first centrifuged at 14,100× *g* for 45 min to precipitate. 500 µL of non-imprinted nanohydrogel (980 mg·mL^−1^) and 500 µL of albumin-imprinted nanohydrogel (990 mg·mL^−1^) remaining in the precipitated phase were separately added to 1000 µL of albumin solution prepared in artificial urine samples within the range of 0–300 ppm. After the mixtures showed a homogeneous distribution, fluorescence measurements were taken.

## 3. Results and Discussion

### 3.1. Characterisation Analysis

**Figure 2:**
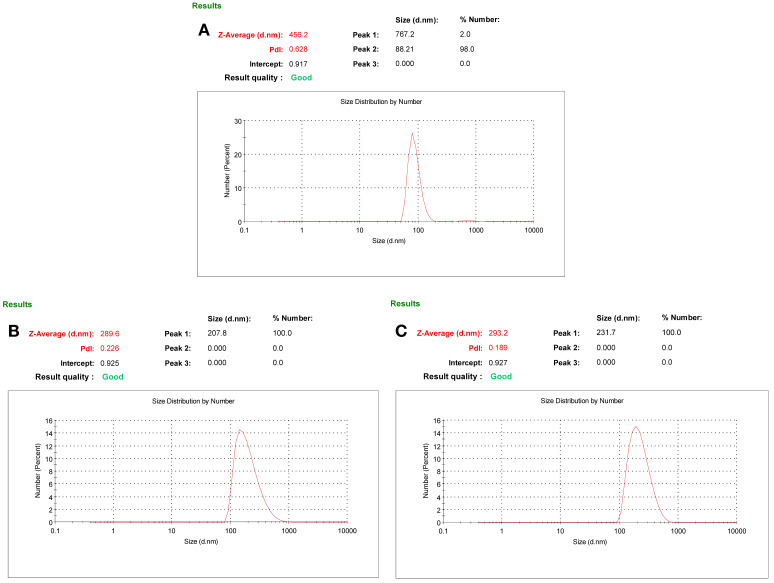
The zeta size analysis of (**A**) C-Dots, (**B**) non-imprinted and (**C**) albumin-imprinted nanohydrogels.

Measurements performed using ZetaSizer provide important information regarding the hydrodynamic diameters and size distribution quality of synthesised nanoparticles [32]. These analyses are critical for evaluating the homogeneity of the system, as they reveal both particle sizes and polydispersity index (PDI) values [33]. In the zeta size analysis of the C-Dots shown in Figure 2A, the particle size was measured to be 88.21 nm. This value confirms that C-Dots are small and uniform structures. Their small size increases the surface area, enhancing their interaction capacity with target biomolecules such as albumin. The zeta size analysis of non-imprinted nanohydrogels shown in Figure 2B measured with size of 200.8 nm. Considering that these structures were synthesised without an imprinted molecule, albumin, it can be said that the pore structure is less pronounced and the internal volume is more limited. The increase in size is related to the swelling behaviour and structural density of the hydrogel matrix. The particle size measured in the zeta size analysis of albumin-imprinted nanohydrogels shown in Figure 2C was 231.7 nm. This increase is due to albumin acting as a template molecule, which creates specific recognition sites within the matrix. The size expansion in these structures indicates that the hydrogel network structure has evolved into a more porous and target-responsive form. This represents a key advantage, enhancing selective binding capacity and allowing all results to be considered high-quality. This indicates that the molecular imprinting process creates a larger and more target molecule-sensitive pore structure in the nanohydrogel, which can increase albumin binding capacity.

**Figure 3:**
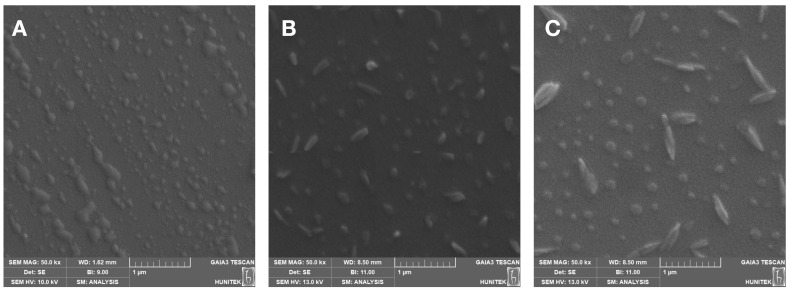
The SEM images of (**A**) C-Dots, (**B**) non-imprinted and (**C**) albumin-imprinted nanohydrogels.

SEM is a widely used imaging technique for examining the morphological properties of nanomaterial surfaces at high resolution. Signals obtained from the sample surface using an electron beam enable detailed analysis of particle size, shape and surface topography [34]. According to the SEM images of C-Dots shown in Figure 3A, these structures have a spherical morphology and consist of relatively uniform morphology and distribution particles. The particle size is approximately ~80 nm. This result is consistent with zeta size analyses. This structure demonstrates that C-Dots possess effective binding capabilities in biomedical applications due to their high surface area and good dispersion properties. According to the SEM images of non-imprinted nanohydrogels given in Figure 3B, the structure of nanohydrogels synthesised without using an albumin template has a relatively spherical morphology and consists of uniform morphology and distribution particles. The particle size is approximately ~200 nm, which is consistent with zeta size analyses. The capacity of these structures to recognise or selectively bind large biomolecules, such as albumin, is limited because they lack specific recognition sites. This indicates that binding is limited to weak forces such as physical adsorption or hydrophobic interactions. Furthermore, compared with albumin-imprinted nanohydrogels, non-imprinted nanohydrogels have lower pore volume and surface area, thereby significantly reducing bonding efficiency. The SEM images of albumin-imprinted nanohydrogels shown in Figure 3C reveal a notable volumetric increase in the spherical structure, highlighting the effectiveness of the albumin-imprinting method in enhancing their characteristics. This porous structure clearly demonstrates that the hydrogel network contains specific binding sites for albumin and that the imprinting process was successful. Furthermore, the enhanced surface area of albumin-imprinted nanohydrogels supports their binding kinetic dynamics. This morphology of the albumin-imprinted structure demonstrates that it is both functionally advanced and more advantageous for bioseparation applications compared to the non-imprinted structure. On average, a particle size of 250 nm is consistent with zeta size analyses. The uniform morphology and distribution of particles observed in SEM images indicate that the nanohydrogels were obtained through a homogeneous polymerization process. These morphological features contribute to the greater accessibility of the recognition sites to the surface, positively influencing the interaction kinetics with the target protein.

**Figure 4:**
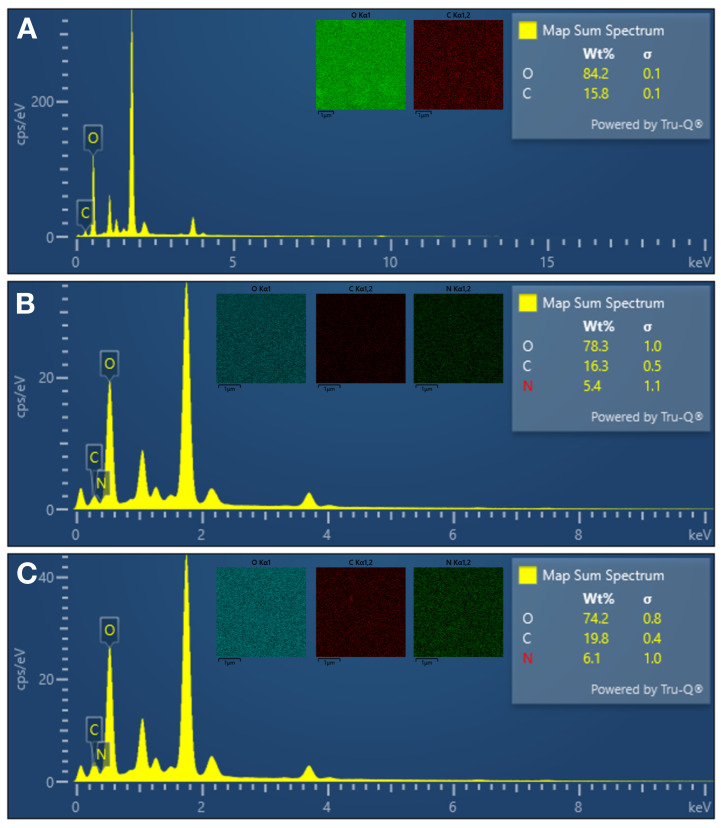
The EDX analysis of (**A**) C-Dots, (**B**) non-imprinted and (**C**) albumin-imprinted nanohydrogels.

EDX is a powerful technique widely used for the quantitative and qualitative analysis of the elemental composition of nanostructures [35]. The EDX analyses conducted in this study were performed to comparatively evaluate the surface compositions of C-Dots and non-imprinted and albumin-imprinted nanohydrogels. In the EDX spectrum of the C-Dots shown in Figure 4A, the signals belonging to the elements carbon (C) and oxygen (O) are clearly observed. This result confirms that C-Dots have a carbon-based structure and contain oxygen-containing functional groups (carboxyl, hydroxyl, etc.) on their surface. These groups are important structures that facilitate the solubility of C-Dots in water and their interaction with biological systems. The EDX spectrum of the non-imprinted nanohydrogels shown in Figure 4B revealed the presence of nitrogen (N) in addition to C and O elements. The presence of nitrogen stems from nitrogen-containing compounds incorporated into the structure, such as leucine methacrylamide (MALM). The nitrogen content indicates that the nanohydrogel structure has the potential to form hydrogen bonds and electrostatic interactions with proteins. Similarly, the EDX spectrum of albumin-imprinted nanohydrogels shown in Figure 4C revealed the presence of C, O and N elements. However, a relatively higher nitrogen ratio was observed in this structure. This corresponds to the amino groups and peptide bonds in the specific recognition sites formed by the albumin template during the imprinting process. Furthermore, this increased nitrogen content supports the presence of functional groups that confer higher selectivity and binding capacity towards the target molecule, albumin. EDX analysis confirms the presence of functional groups containing carbon, oxygen and nitrogen in the nanohydrogel structure, along with the observed elemental distribution. These functional groups facilitate the formation of various weak bonds with protein molecules, such as hydrogen bonding, electrostatic interactions and dipole–dipole interactions, thereby supporting selective interaction with albumin.

**Figure 5:**
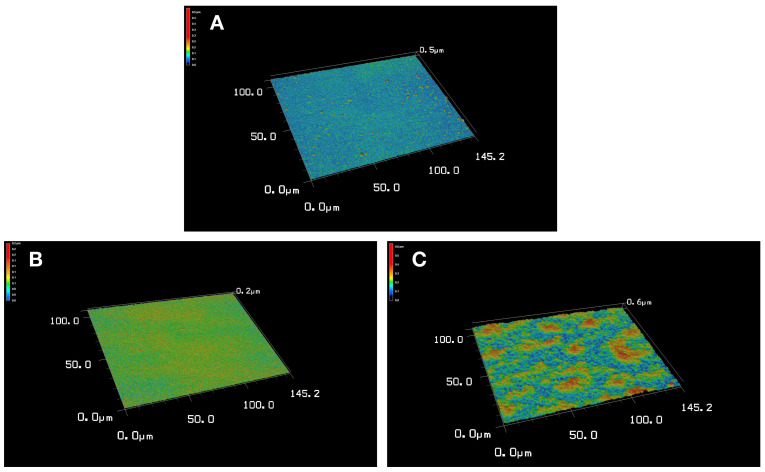
CLSM image of (**A**) C-Dots, (**B**) non-imprinted and (**C**) albumin-imprinted nanohydrogels.

CLSM is a powerful microscopic characterisation technique for three-dimensional imaging and detailed analysis of surface topography [36]. The C-Dots CLSM image shown in Figure 5A demonstrates that the carbon dots have a homogeneous distribution and do not exhibit aggregation. The balanced image across the surface indicates that the C-Dots have been successfully synthesised. This property indicates that C-Dots can be effectively used as signal-generating units in sensor systems and possess high surface activity capable of interacting with biomolecules. The non-imprinted nanohydrogel CLSM image shown in Figure 5B indicates a more compact and less porous surface morphology. The low roughness of the surface indicates that, despite the presence of C-Dots within the nanohydrogel matrix, the structure lacks target molecule-specific recognition pockets. This supports that the interaction with albumin is largely limited to physical adsorption and weak surface interactions and that selective binding does not occur. The CLSM image of the albumin-imprinted nanohydrogel shown in Figure 5C exhibits a distinctly more porous, irregular but homogeneously distributed surface topography. The rougher surface structure indicates that the C-Dots are effectively integrated into the nanohydrogel matrix and that the molecular imprinting process has successfully created recognition pockets specific to the albumin molecule. This porous morphology observed throughout the three-dimensional structure increases the contact surface area with albumin, thereby enhancing binding efficiency and clearly demonstrating the system’s selectivity. These results confirm the success of the molecular imprinting process and that the albumin-imprinted nanohydrogel offers a superior structure for bioselective sensing applications.

**Figure 6:**
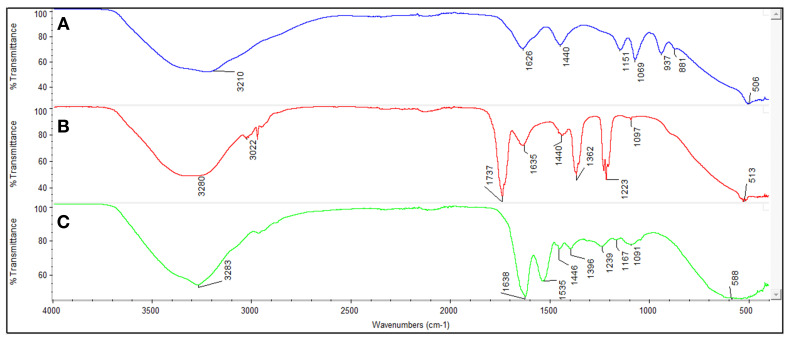
The ATR-FTIR spectrum of (**A**) C-Dots, (**B**) non-imprinted and (**C**) albumin-imprinted nanohydrogels.

ATR-FTIR spectroscopy is a widely used analytical method for identifying the chemical functional groups of materials and verifying whether the synthesis process has been successful [37]. In this study, the chemical structures of C-Dots and non-imprinted and albumin-imprinted nanohydrogels were analysed comparatively. In the ATR-FTIR spectrum of C-Dots shown in Figure 6A, a strong absorption in the 3200–3500 cm^−1^ region, seen as a broad band, indicates -OH (hydroxyl) groups. These groups increase the solubility of C-Dots in water and support their compatibility with biological environments. The sharp peak observed in the 1700–1750 cm^−1^ region confirms the presence of C=O (carbonyl) groups. These groups represent both the stability of the structure and potential interaction regions. Peaks related to C=C (aromatic) and C-H (aliphatic) groups were also observed, demonstrating the structural integrity of C-Dots. In the ATR-FTIR spectrum of the non-imprinted nanohydrogels shown in Figure 6B, the characteristic C=O stretching band of ester groups is prominent around ~1720 cm^−1^. This indicates that monomers such as HEMA and MALM successfully form the hydrogel structure. The bands in the ~3400 cm^−1^ region represent the -OH groups present in the structure. The C-H stretching vibrations between 2800 and 3000 cm^−1^ indicate the aliphatic character of the hydrogel backbone. This analysis proves that the basic hydrogel structure has been successfully formed despite the absence of an albumin template. In the ATR-FTIR spectrum of albumin-imprinted nanohydrogels given in Figure 6C, bands like those in the spectrum of non-imprinted nanohydrogels are observed, but the signals belonging to C=O and N-H groups are noticeably sharper and more intense. In particular, the amide I band observed around ~1650 cm^−1^ indicates the presence of the albumin template. The amide II band, which can be observed between 1550 and 1580 cm^−1^, represents N-H bending vibrations specific to the protein structure. These signals confirm that albumin-specific cavities (recognition sites) remain in the structure after the imprinting process and that molecular imprinting has been successfully achieved. Furthermore, the integration of C-Dots into the structure has increased the spectral intensity of the C=O and O-H groups, indicating enhanced surface functionality.

### 3.2. Optical Analysis

The effect of albumin concentrations of 0, 30, 100, 200 and 300 ppm on the optical analysis of C-Dots, non-imprinted and albumin-imprinted nanohydrogels was investigated. These values encompass the albumin values in human urine in a healthy individual (<30 μg/mL) [38] and the range values in patients with kidney damage and diabetic nephropathy (>30–300 μg/mL) [39].

**Figure 7:**
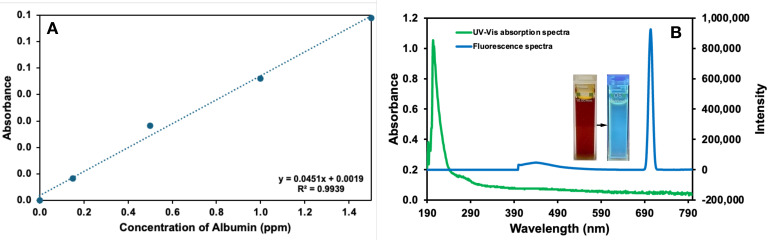
(**A**) Calibration curve obtained from absorbance values of albumin concentrations (0, 30, 100, 200 and 300 ppm) diluted at a ratio of 1:200 (*v*/*v*), (**B**) UV-Vis absorption spectrum of C-Dots diluted at a ratio of 1:200 (*v*/*v*) (green line), fluorescence spectrum of C-Dots diluted at a ratio of 1:1000 (*v*/*v*) (blue line), images of C-Dots under white light (light brown) and UV light (bright blue). Experimental conditions: T = 25 °C; λ_exi_: 350 nm; Slit Width: EX: 5.0 nm, EM: 5.0 nm.

Figure 7A shows the calibration curve plotted using the absorbance values obtained at a wavelength of 280 nm for different albumin concentrations. The calibration curve obtained showed a strong linear relationship (R^2^ = 0.99) between the absorbance values and the albumin concentration. The absorption observed in the 280 nm region is due to the characteristic absorption of the aromatic amino acids (tryptophan, tyrosine) present in the structure of albumin. The obtained linear relationship demonstrates that the system operates with high accuracy and reproducibility over a wide concentration range. The linear increase in the calibration graph demonstrates the proportional effect of albumin concentration on optical density and proves the system’s suitability for quantitative determination. This result demonstrates that the binding efficiency of the developed C-Dots-modified nanohydrogels and the interaction of albumin with the system can be spectroscopically monitored. In particular, the linear behaviour in the 0–300 ppm range supports the potential of this method as a biosensor basis for detecting microalbuminuria levels. Figure 7B shows the UV-Vis absorption and fluorescence spectra of C-Dots diluted at a 1:1000 (*v/v*) ratio, along with their images under white light and UV light. The distinct absorption band typically observed in the UV-Vis absorption spectrum between ~270–340 nm represents π-π* transitions (C=C bonds) and *n*-π* transitions (C=O groups). These bands confirm the presence of oxygen- and nitrogen-containing functional groups on the surface of C-Dots. The strong emission peak observed in the fluorescence spectrum indicates that C-Dots possess high quantum yield and excellent photostability. This property demonstrates that C-Dots are advantageous for optical tracking and sensor applications in biological systems. In the images captured, the C-Dots solution appears light brown under white light and bright blue under UV light. This confirms that the synthesised C-Dots have successfully acquired fluorescent properties and that the surface functionalisation is effective.

**Figure 8:**
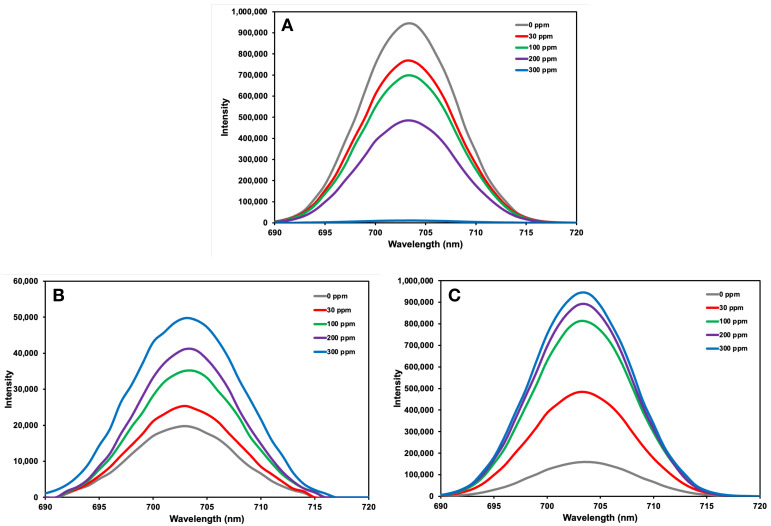
The effect of albumin concentration on fluorescence spectra of (**A**) C-Dots, (**B**) non-imprinted and (**C**) albumin-imprinted nanohydrogels. The experimental conditions: pH = 7.4; T = 25 °C; λ_exi_: 350 nm; Slit Width: EX: 5.0 nm, EM: 5.0 nm.

In the fluorescence spectra of C-Dots shown in Figure 8A, a significant decrease in the fluorescence intensity of C-Dots was observed with increasing albumin concentration. This can be explained by the interaction of albumin molecules with active sites on the surface of C-Dots, creating a quenching effect. The decrease in fluorescence intensity observed with the addition of albumin molecules to the C-Dots solution is due to the interactions between albumin and the C-Dots surface. This is particularly associated with π–π* interactions and surface adsorption between the aromatic amino acids of albumin and the π-systems on the C-Dots surface. Furthermore, the adsorption of albumin molecules to the C-Dots surface reduces the distance between C-Dots particles, contributing to a self-quenching mechanism. The interaction of C-Dots with such proteins enables the use of fluorescence changes as an analytical sensor. In the fluorescence spectra of non-imprinted nanohydrogels shown in Figure 8B, the fluorescence intensity, which increases in parallel with the increase in albumin concentration, is generally low. This indicates that binding occurs primarily through physical adsorption, as the non-imprinted nanohydrogels do not contain recognition sites. In the fluorescence spectra of albumin-imprinted nanohydrogels shown in Figure 8C, the increase in fluorescence intensity parallel to the increase in albumin concentration is more pronounced and significant. The different optical behaviour is observed when C-Dots are located within an albumin-imprinted nanohydrogel structure. Albumin molecules tend to bind to specific recognition voids within the nanohydrogel matrix. This binding limits the direct interaction of albumin with the C-Dots surface, reducing the fluorescence quenching effect observed in the free C-Dots system. Furthermore, the binding of albumin to recognition sites can contribute to an increase in the fluorescence signal by altering the environmental microenvironment of C-Dots within the nanohydrogel. Therefore, a significant increase in the fluorescence signal is observed with increasing albumin concentration in albumin-imprinted nanohydrogels. This increase indicates that albumin binds more effectively to the surface of albumin-imprinted nanohydrogels due to specific recognition sites and that this binding affects the fluorescence properties of C-Dots.

### 3.3. Selectivity Studies

**Figure 9:**
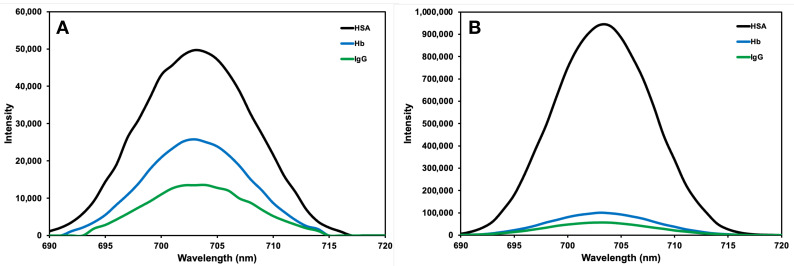
Comparison of the effect of 300 ppm HSA, Hb and IgG on the fluorescence spectra of (**A**) non-imprinted and (**B**) albumin-imprinted nanohydrogels. The experimental conditions: C_protein_ = 300 ppm; pH = 7.4; T = 25 °C; λ_exi_: 350 nm; Slit Width: EX: 5.0 nm, EM: 5.0 nm.

To evaluate the selectivity behaviour of the developed nanohydrogels, their interactions with HSA, Hb and IgG molecules were analysed using fluorescence spectroscopy. The results presented in Figure 9 show the fluorescence spectra obtained from the interactions of both albumin-imprinted and non-imprinted nanohydrogels with these molecules. In non-imprinted nanohydrogels (Figure 9A), although the albumin peak was the highest, the fluorescence intensities observed with Hb and IgG were closer to each other. This finding indicates that, due to the absence of specific recognition sites in the non-imprinted structure, binding is limited mainly to physical adsorption and surface interactions. The significantly higher fluorescence response observed for albumin in the imprinted structure compared to other molecules clearly demonstrates that nanohydrogels exhibit high selectivity and specific binding capacity. In albumin-imprinted nanohydrogels (Figure 9B), the fluorescence peak intensity, observed as a result of interaction with albumin, is significantly higher compared to Hb and IgG. This indicates that the specific recognition sites formed during synthesis for albumin were successfully created and that the imprinted structure recognises the target molecule with high specificity. The strong peak observed for albumin proves that the spaces specifically formed for the albumin molecule within the nanohydrogel matrix provide effective binding. The molecular imprinting process shows that the recognition cavities formed within the nanohydrogel matrix are compatible with the size, shape and functional group characteristics of the albumin molecule. Therefore, stronger and more specific interactions are formed with albumin compared to competing proteins, and the analytical selectivity of the system is increased. The lower fluorescence responses obtained in interactions with Hb and IgG are due to differences in the molecular size, surface charge and isoelectric points of these proteins. This situation reveals that the immobilised structure interacts not only through general electrostatic attraction but also through specific binding based on molecular recognition.

The selectivity coefficients (k), relative selectivity coefficients (k’) and imprinting efficiency (IE) provided in Table 1, given that albumin-imprinted nanohydrogels exhibit high specificity towards the target protein albumin. The calculated k’ = 4.87 for Hb and k’ = 4.55 for IgG in the albumin-imprinted nanohydrogels indicate a significant increase in albumin recognition capacity compared to the non-imprinted nanohydrogels. These results confirm that the specific recognition sites created by the imprinting process function successfully and that the composite nanohydrogels exhibit high performance in terms of selectivity. The calculated IE value (18.96) indicates that the albumin-specific recognition regions have been successfully formed in the albumin-imprinted nanohydrogel structure and that the albumin binds to these structures more effectively than to non-imprinted structure. Therefore, compression efficiency is a critical evaluation criterion that directly reflects the selectivity and molecular recognition performance of the developed nanohydrogel structure.

### 3.4. Repeatability and Long-Term Stability Studies

**Figure 10:**
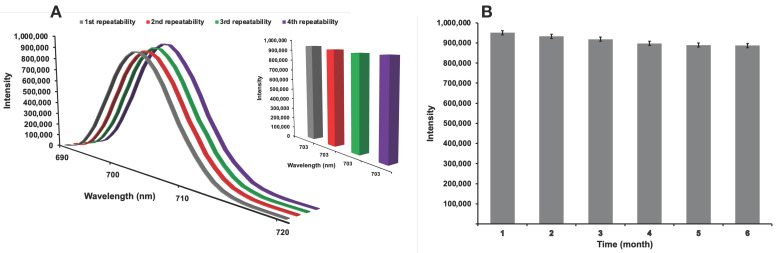
Repeatability and long-term stability studies of albumin-imprinted composite nanohydrogel: (**A**) fluorimetric analyses of four consecutive measurements performed with 300 ppm albumin solution, comparison of the maximum fluorescence intensity values obtained from these fluorimetric analyses and (**B**) fluorimetric analyses of long-term stability measurements (six month) performed with 300 ppm albumin solution. The experimental conditions: pH = 7.4, T = 25 °C.

For albumin detection, four consecutive repeatability and six-month long-term stability fluorescence measurements were performed using an albumin-imprinted nanohydrogel with a 300 ppm albumin solution. This analysis is given that in Figure 10. The variation between the obtained fluorescence intensities was evaluated and the %RSD value was calculated to express the statistical consistency of the measurements. The calculated low %RSD value (%RSD < 1.5) indicates negligible deviation between measurements and demonstrates the high measurement stability of the system. These results demonstrate that the albumin-imprinted nanohydrogel maintains its structural integrity and binding capacity during interaction with albumin and that no significant performance loss occurs in repeated measurements. Therefore, it has been confirmed that the developed nanohydrogel-based system can be used as a reliable, stable and reproducible detection platform in biological analyses.

### 3.5. Analysis in Artificial Urine Samples

**Figure 11:**
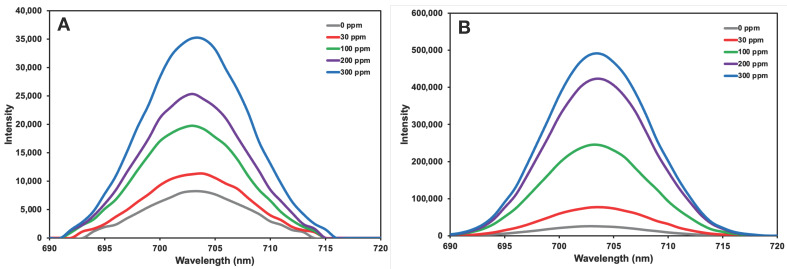
The effect of albumin concentration in artificial urine samples on fluorescence spectra of (**A**) non-imprinted and (**B**) albumin-imprinted nanohydrogels. The experimental conditions: T = 25 °C.

Experimental studies conducted to evaluate the performance of hybrid nanohydrogels developed in a system resembling real biological environment conditions examined the fluorescence spectra using artificial urine samples containing albumin at different concentrations. As shown in Figure 11, the fluorescence spectra obtained in the artificial urine environment are generally lower in intensity compared to measurements made in PBS buffer. It can be due to the matrix effect caused by the presence of urea, creatinine, inorganic salts (NaCl, NH_4_Cl, CaCl_2_, Na_2_HPO_4_) and organic compounds naturally found in artificial urine. Such components may have partially impeded the interaction of albumin with the recognition sites on the nanohydrogel surface, thereby reducing the binding efficiency of albumin and the intensity of the fluorescence spectra. Furthermore, the increase in ionic strength in the solution may have enhanced fluorescence quenching by weakening electrostatic interactions. However, in albumin-imprinted nanohydrogels, a higher fluorescence response for albumin was observed in artificial urine, as in PBS buffer, compared to the non-imprinted nanohydrogels. This demonstrates that the specific recognition sites formed during the imprinting process function effectively even in complex biological environments such as artificial urine. This provides a significant advantage in terms of the applicability of the developed system to real biological samples. Lower signal intensities were obtained in non-imprinted nanohydrogels. This confirms that the binding is largely based on physical adsorption and that no specific interactions are present.

Hydrogel-based sensing systems containing fluorescent nanomaterials have become an important research area in recent years. Li et al. developed a fluorescence-based biosensor that can detect haemoglobin with high sensitivity and selectivity by combining hydrogel nanoparticles with C-Dots [30]. Mohammadi et al. designed a hydrogel biosensor based on C-Dots-chitosan and detected microRNA-21 in breast cancer cells by fluorescence with very low detection limits [40]. Chen et al. developed an environmentally friendly system that can both detect and adsorb Cu(II) and Cr(VI) heavy metal ions by fluorescence by developing a C-Dots cross-linked hydrogel containing cellulose nanofibrils and chitosan [41]. Bhattacharya et al. developed a sensor that detects reactive oxygen species (ROS) with high sensitivity by means of fluorescence extinction mechanism with C-Dots embedded in an ascorbic acid-based hydrogel [42]. In another study, Bhattacharya et al. demonstrated that bacterial detection can be achieved based on the principle of destruction of the C-Dots hydrogel hybrid structure due to enzymes secreted by bacteria and extinguishing of fluorescence [43]. However, in most of these studies, the detection mechanism is based on non-specific interactions with the target analyte. The C-Dots-modified molecularly imprinted nanohydrogel system developed in this study offers selective binding sites for albumin with specific recognition cavities created within the hydrogel matrix. Thus, by combining the strong fluorescence properties of C-Dots with the selectivity of the molecular imprinting technology, a fast, selective, label-free and ultra-sensitive detection platform for albumin detection has been obtained.

## 4. Conclusions

In this study, C-Dots-modified composite nanohydrogel structures were successfully developed using molecular imprinting technology for the rapid, ultra-sensitive, selective and label-free detection of albumin in human urine. C-Dots fluorescent probes and albumin-imprinted nanohydrogels were used as sensing materials. The results obtained revealed that the synthesised albumin-imprinted nanohydrogel systems exhibited high performance not only structurally but also functionally. In particular, the albumin-imprinted structures showed significant superiority in terms of binding capacity, selectivity and signal stability compared to non-imprinted nanohydrogels. SEM, CLSM, zeta size and EDX analyses confirmed that albumin-imprinted nanohydrogels exhibit a more porous structure with a larger surface area and contain albumin-specific recognition sites. The distinct Amide I and Amide II bands observed in ATR-FTIR analyses proved that albumin-specific recognition pockets were successfully formed within the polymer matrix as a result of the molecular imprinting process. These structural features formed the basis for specific interactions with albumin. Fluorometric analyses showed that the fluorescence signal obtained in albumin-imprinted nanohydrogel systems increased significantly as the albumin concentration increased. This revealed that the integration of C-Dots into the structure not only increased optical traceability but also contributed to stable signal generation sensitive to albumin binding. Selectivity studies determined that albumin-imprinted nanohydrogels exhibit significantly higher selectivity towards albumin compared to competitive molecules, Hb and IgG. The calculated high relative selectivity coefficients and suppression efficiency value clearly demonstrate the effectiveness of the molecular imprinting process. The low %RSD value obtained in the reproducibility studies demonstrated that the developed system offers high stability and reliability in sequential measurements. Furthermore, in analyses conducted in artificial urine media, it was observed that although the complex biological matrix reduced the signal intensity to some extent, the albumin-imprinted nanohydrogels retained their selective recognition capabilities. These results demonstrate that the developed system can function effectively not only in ideal buffer media but also in biologically complex environments. The C-Dots-modified albumin-imprinted composite nanohydrogel systems developed in this study offer a promising platform for urine-based albumin detection with their high sensitivity, selectivity, reproducibility and applicability in biological environments. These results demonstrate that the developed C-Dots-modified albumin-imprinted nanohydrogels provide a promising platform for sensitive and selective albumin detection in complex biological environments.

## Figures and Tables

**Table 1:** The intensity values (I), selectivity coefficients (k), relative selectivity coefficients (k’) and imprinting efficiency (IE) of HSA, Hb and IgG for imprinted and non-imprinted composite nanohydrogels. The experimental conditions: C_protein_ = 300 ppm; pH = 7.4; T = 25 °C; λ_exi_: 350 nm; Slit Width: EX: 5.0 nm, EM: 5.0 nm.

	I_imprinted_	I_non-imprinted_	k_imprinted_	k_non-imprinted_	k’	IE
**HSA**	942.21	49.70	-	-	-	18.96
**Hb**	100.32	25.81	9.39	1.93	4.87	-
**IgG**	56.31	13.52	16.73	3.68	4.55	-

## Data Availability

The data that support the findings of this study are available on request from the corresponding author. The data are not publicly available due to privacy or ethical restrictions.

## Supplementary Materials

The following supporting information can be downloaded at: https://www.mdpi.com/article/10.3390/mi17040409/s1, Figure S1. The fluorescence spectra of non-imprinted (green) and template-removed albumin-imprinted nanohydrogels (blue). The experimental conditions: pH = 7.4, T = 25 °C.
