# Supplementary material for: Ultrasensitive Human Urinary Albumin Detection via Composite Nanohydrogels
Source: Micromachines (Basel). 2026 Mar 27;17(4):409. doi: 10.3390/mi17040409 (PMC13117906; doi:10.3390/mi17040409)
Supplement: Supplementary file 1 [file micromachines-17-00409-s001.zip › micromachines-4166544-supplementary.pdf]

Supplementary Materials: Ultrasensitive Human Urinary Albumin Detection via Composite Nanohydrogels

Özge Altıntaş [1], Fatma Yılmaz [2], Elif Serra Taş [3] and Adil Denizli [1,*]

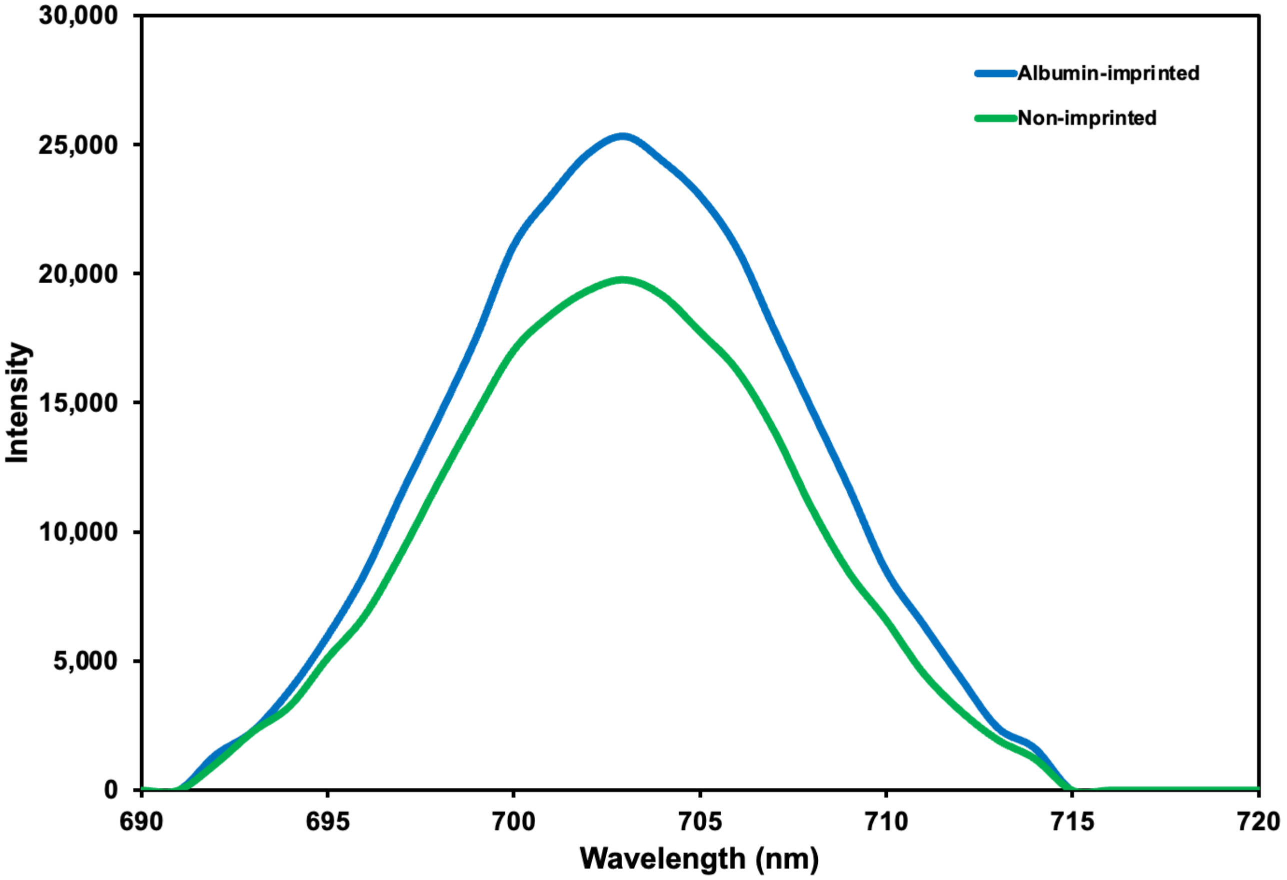

Figure S1. The fluorescence spectra of non-imprinted (green) and template-removed albu-min-imprinted nanohydrogels (blue). The experimental conditions: pH = 7.4, T = 25 °C.

Disclaimer/Publisher's Note: The statements, opinions and data contained in all publications are solely those of the individual author(s) and contributor(s) and not of MDPI and/or the editor(s). MDPI and/or the editor(s) disclaim responsibility for any injury to people or property resulting from any ideas, methods, instructions or products referred to in the content.
